# Asymmetric dual-process mechanisms under symbolic exposure: music-induced destination perception

**DOI:** 10.3389/fpsyg.2026.1891404

**Published:** 2026-10-12

**Authors:** Xiasui Peng, Bao-Hui Huang, Cheng-Ru Wu, Qin Su

**Affiliations:** 1 College of Tourism Management, Wuyi University, Wuyishan, China; 2 College of Geography and Tourism, Anhui Normal University, Wuhu, China

**Keywords:** behavioral intentions, cognitive evaluation, destination songs, music-evoked emotion, place attachment, symbolic exposure

## Abstract

**Background:**

Music connects listeners with places through imagery, cultural meanings, and personal associations. How cognitive and emotional responses relate to place attachment and behavioral intentions in destination-song contexts remains unclear.

**Objective:**

This study examines cognitive–affective pathway differences and the relational role of place attachment.

**Methods:**

A mixed-methods design combined Latent Dirichlet Allocation analysis of 57,228 NetEase Cloud Music comments on 15 destination-related songs with a survey of 472 respondents. Expert interpretation and psychological theory informed construct development. PLS-SEM assessed direct and indirect associations, with 5,000 paired bootstrap resamples used for exploratory pathway comparisons.

**Results:**

Place imagery, local cultural meaning, and associative experience were positively associated with cognitive evaluation and emotional experience. Pathway comparisons showed stronger associations between place imagery and cognitive evaluation, and between associative experience and emotional experience. Cognitive–affective differences in paths to attachment, visit intention, and sharing intention were not significant. Place attachment partially mediated the associations of cognitive evaluation (β = 0.040, 95% CI [0.016, 0.072]) and emotional experience (β = 0.050, 95% CI [0.021, 0.086]) with visit intention.

**Conclusion:**

The findings refine the SOR framework by locating asymmetry in selected antecedent pathways and identifying place attachment as a shared relational link to visit intention. The cross-sectional design and predominance of previous visitors limit conclusions about causal ordering and new attachment formation.

## 1 Introduction

Digital media provide access to destination information and place narratives through images, sound, and text. Streaming platforms, social media, and user-generated content (UGC) offer material through which people understand and imagine destinations (Guo and Pesonen, 2022; Pop et al., 2022; Abbasi et al., 2023). Destination perception can therefore draw on mediated cues alongside direct experience.

Destination songs connect musical experiences with places through melodies, lyrics, emotional tone, and cultural narratives (Bassano et al., 2019; Bennett and Janssen, 2016; Zhuang et al., 2023). Music psychology has examined how listening evokes emotions, autobiographical memories, and imagination (Juslin et al., 2014; Koelsch, 2014; Eskine et al., 2020; Margulis and Jakubowski, 2024), while destination-song research has documented associations with destination image and visit intention (Yin et al., 2020; Lv et al., 2022; Wei et al., 2023). These studies provide a basis for examining how emotional engagement with a song relates to evaluations of, and psychological bonds with, the represented place. Such mediated encounters may involve both imagined experiences and meanings grounded in previous visits.

Clarifying these relationships requires distinguishing emotional experience, cognitive evaluation, and place attachment. Tourism research has linked emotion, image, and nostalgia with tourist responses (Prayag et al., 2017; Ramkissoon, 2023; Lu et al., 2022; Wang, 2023; Bhogal et al., 2024), and examined how electronic word of mouth, mediated information, and prior experience relate to evaluations and intentions (Jalilvand and Samiei, 2012; Guo and Pesonen, 2022; Abbasi et al., 2023; Hu and Xu, 2021). Musical emotions also vary with personal experience and listening context (López et al., 2024; Juslin, 2025). Feeling moved by a song, evaluating a destination favorably, and feeling attached to it are therefore conceptually distinct. Further examination is needed of their connections: whether attachment links cognitive and emotional responses with visit intention, how these responses relate to sharing intention, and where corresponding cognitive–affective pathways differ in strength. This approach distinguishes intended travel from communication about songs and destinations without presuming cognitive or emotional dominance.

Although analytically distinct, emotional responses, personal memories, and place-related meanings may intertwine in listeners’ accounts of musical experiences (Margulis and Jakubowski, 2024). UGC captures these expressions in context, providing material for connecting psychological concepts with the language listeners use (Wang et al., 2024). Latent Dirichlet Allocation (LDA) can identify recurring semantic themes across large comment corpora (Blei et al., 2003), while theory-guided interpretation helps assess their relevance to psychological constructs (Maier et al., 2018). Combining this semantic exploration with survey measurement allows listeners’ expressions to inform construct contextualization and supports the examination of individual-level psychological associations.

Accordingly, this study combines LDA and partial least squares structural equation modeling (PLS-SEM), drawing on music psychology, tourism psychology, and the Stimulus–Organism–Response (SOR) framework. It examines how place imagery, local cultural meaning, and associative experience relate to cognitive evaluation and emotional experience, and how these responses connect with place attachment, visit intention, and sharing intention. Additional exploratory comparisons assess differences between corresponding cognitive and affective pathways. The study seeks to clarify where these pathways differ, how attachment connects them with visit intention, and how travel-oriented intentions relate to communicative engagement.

## 2 Literature review and hypothesis development

### 2.1 Destination songs as symbolic environmental stimuli

Destination songs serve as symbolic environmental stimuli by representing places through melodies, lyrics, emotional tone, and culturally embedded narratives (Bassano et al., 2019; Bennett and Janssen, 2016; Moreno-Lobato et al., 2023). Here, symbolic exposure refers to encountering a destination through its mediated representation, regardless of prior visitation. Although photographs and written narratives also convey place meanings, destination songs combine musical expression with place-related references, inviting listeners to interpret these references through imagination and personal associations (McAuley et al., 2021; Margulis and Jakubowski, 2024; Taruffi et al., 2017). This combination helps explain the relevance of music to destination promotion and mediated tourism communication (Coronel and Irimiás, 2023).

Music listening can involve emotional contagion, autobiographical memory, imagery, and self-related interpretation (Juslin et al., 2014; Koelsch, 2014; Schäfer et al., 2013). These experiences provide a basis for considering how listeners mentally engage with represented places beyond immediate physical presence (Eskine et al., 2020; Herff et al., 2021). Tourism studies have linked music and destination-song perceptions to destination image and behavioral intentions (Yin et al., 2020; Lv et al., 2022; Wei et al., 2023; Min et al., 2019). Research on UGC further highlights the role of mediated content in destination imagery and audience responses (Guo and Pesonen, 2022; Abbasi et al., 2023; Aboalganam et al., 2025). Together, these perspectives suggest that destination songs connect musical engagement with place-related interpretation through meanings that listeners bring to the listening experience.

These listener-dependent meanings help explain why the same song may evoke different responses. Musical interpretation varies with mood, personality, autobiographical relevance, prior experience, and listening context (Vuoskoski and Eerola, 2011; López et al., 2024), producing diverse emotional experiences across listeners (Warrenburg, 2020; McCrae, 2021). A song’s emotional tone therefore cannot be equated with the listener’s feelings or evaluation of the destination, nor should these responses be assumed to be uniformly positive. This distinction motivates examining cognitive evaluation and emotional experience separately and assessing their associations with attachment and behavioral intentions.

### 2.2 Music-induced psychological processing under symbolic exposure

Building on the variability outlined above, understanding destination-song responses requires distinguishing how listeners interpret a represented place from how they feel during listening. Tourism research has examined relationships among emotional experience, destination evaluations, and relational or behavioral outcomes (Prayag et al., 2017; Ramkissoon, 2023). Under symbolic exposure, listeners may draw on imagined scenes, autobiographical associations, and narrative engagement to make sense of the destination (McAuley et al., 2021; Margulis and Jakubowski, 2024; Taruffi et al., 2017; Green and Brock, 2000). These sources of meaning may inform both destination judgments and emotional involvement, providing a basis for examining cognitive and affective responses as related but distinct.

Cognitive evaluation concerns judgments of a destination’s attractiveness and desirability, informed by the interpretation of mediated information and place meanings (Jalilvand and Samiei, 2012; Guo and Pesonen, 2022; Abbasi et al., 2023; Aboalganam et al., 2025). Emotional experience concerns emotional intensity and involvement during listening, drawing on the music-evoked processes discussed above (Juslin et al., 2014; Koelsch, 2014; Schäfer et al., 2013). Research on music-related imagination provides a basis for examining how these listening experiences connect listeners with represented destinations (Hashim et al., 2023; Herff et al., 2021). These responses may overlap, but they differ in focus: cognitive evaluation addresses the destination, whereas emotional experience captures feelings during engagement with the song.

Cognitive evaluation and emotional experience may both relate to place attachment, but these associations do not establish when attachment emerged or how long it persists. Musical emotions vary with personal relevance, mood, and listening context (Vuoskoski and Eerola, 2011; López et al., 2024; Juslin, 2025), and emotional intensity alone does not specify the meaning of the experience (Warrenburg, 2020; McCrae, 2021). These distinctions provide a basis for examining whether cognitive and affective responses differ in their antecedents and associations with attachment and behavioral intentions, without assuming temporal precedence or a fixed hierarchy.

### 2.3 SOR and asymmetric dual-process mechanisms

The Stimulus–Organism–Response (SOR) framework links environmental stimuli to behavioral responses through internal psychological states (Mehrabian and Russell, 1974), organizing relationships among evaluations, emotions, and relational outcomes in tourism (Prayag et al., 2017; Ramkissoon, 2023). Here, place imagery, local cultural meaning, and associative experience represent perceived symbolic cues; cognitive evaluation, emotional experience, and place attachment represent internal responses; and visit and sharing intentions represent behavioral intentions. Attachment provides a potential relational link between cognitive and emotional responses and visit intention.

Dual-process perspectives distinguish interacting rational and experiential processing (Epstein, 1994) and emphasize specifying their interaction (De Neys, 2023). Affective preferences need not depend on deliberate inference (Zajonc, 1980), while appraisal theory emphasizes evaluations of personal significance (Lazarus, 1991). Distinctions between adaptive processes and conscious feelings (LeDoux, 2012), alongside cognitive–affective interdependence (Pessoa, 2023), caution against treating destination judgments and listening-related feelings as independent processing systems. These perspectives guide conceptual distinctions; the questionnaire does not test processing speed, automaticity, or temporal precedence.

Mediated information and prior experience can inform destination evaluations and behavioral intentions (Jalilvand and Samiei, 2012; Guo and Pesonen, 2022; Abbasi et al., 2023; Hu and Xu, 2021), while musical engagement varies with personal relevance and listening conditions (Vuoskoski and Eerola, 2011; López et al., 2024; Warrenburg, 2020; Juslin, 2025). Place imagery provides representations for evaluation, associative experience connects listening with personal memories and imagined experiences, and local cultural meaning may inform both interpretation and emotional engagement. These functions motivate comparing each cue’s associations with cognitive evaluation and emotional experience.

Such comparisons also extend to cognitive and emotional associations with attachment, visit intention, and sharing intention. The Framework for Adaptable Musical Emotions highlights the situational, cultural, personal, and psychophysical conditions of musical responses (Susino et al., 2025), offering context for interpreting potential differences without predicting their direction. The study therefore examines asymmetry as differences in the strength of corresponding cognitive and affective associations across antecedent and outcome pathways, without presuming a general hierarchy. Direct comparisons of path coefficients assess these differences; significance within individual pathways alone is insufficient.

### 2.4 Symbolic interpretation and psychological processing

#### 2.4.1 Place imagery, local cultural meaning, and associative experience

Destination songs provide spatial, cultural, and personal references through which listeners interpret represented places (Bassano et al., 2019; Bennett and Janssen, 2016; Zhuang et al., 2023). Place imagery concerns the mental visualization of a destination’s scenes, environment, and atmosphere, drawing on music-related imagination and spatial simulation (Herff et al., 2021; Hashim et al., 2023). Local cultural meaning concerns listeners’ perceptions of a destination’s cultural characteristics and identity, conveyed through dialects, narratives, musical styles, and local references (Bassano et al., 2019; Moreno-Lobato et al., 2023). Associative experience concerns connections between the song, personal memories, life experiences, and imagined involvement with the destination (Juslin et al., 2014; Eskine et al., 2020). These constructs distinguish what listeners imagine about a place, what cultural meanings they perceive, and how they relate the song to themselves. They represent perceived aspects of the listening experience rather than objective musical properties.

#### 2.4.2 Cognitive evaluation

Cognitive evaluation refers to judgments of a destination’s attractiveness and desirability based on the song and its associated meanings (Jalilvand and Samiei, 2012; Guo and Pesonen, 2022). Research on mediated information and prior experience provides a basis for examining how listeners form evaluations relevant to behavioral intentions (Abbasi et al., 2023; Hu and Xu, 2021). Place imagery supplies representations to evaluate, local cultural meaning provides information about destination distinctiveness, and associative experience introduces personal relevance (Yin et al., 2020; Wei et al., 2023; Bassano et al., 2019; Moreno-Lobato et al., 2023; Juslin et al., 2014; Eskine et al., 2020). Cognitive evaluation thus captures an appraisal of the destination, distinct from the vividness of its imagery, emotional involvement during listening, or a personal bond with the place.

#### 2.4.3 Emotional experience and place attachment

Emotional experience refers to emotional intensity and involvement during destination-song listening, which may involve contagion, autobiographical associations, and immersion (Juslin et al., 2014; Koelsch, 2014; Vuoskoski and Eerola, 2011). Place imagery, cultural meaning, and associative experience may support such engagement through nostalgia and personal recollections (Wang, 2023; Bhogal et al., 2024). The construct captures emotional involvement rather than a specific emotion or positive–negative valence (López et al., 2024; Juslin, 2025).

Place attachment concerns emotional connection, personal meaning, and psychological closeness to the represented destination. Tourism research provides grounds for examining its relationship with emotional experience (Prayag et al., 2017; Ramkissoon, 2023; Lu et al., 2022). Given the diversity of musical responses (Warrenburg, 2020; McCrae, 2021), feelings during listening should be distinguished from bonds with the place. These responses may be positively associated, without implying that attachment first emerged during listening.

### 2.5 Hypotheses development

Destination songs provide cues for evaluating represented places. Place imagery makes destination scenes and attributes mentally accessible, while local cultural meaning supports appreciation of their distinctiveness (Yin et al., 2020; Wei et al., 2023; Bassano et al., 2019; Moreno-Lobato et al., 2023). Associative experience may support favorable evaluation by connecting place meanings with personal experiences and imagined involvement (Juslin et al., 2014; Eskine et al., 2020). Accordingly:

H1a. Place imagery is positively associated with cognitive evaluation.

H1b. Local cultural meaning is positively associated with cognitive evaluation.

H1c. Associative experience is positively associated with cognitive evaluation.

These cues may also support emotional engagement. Imagined scenes can be emotionally evocative, while cultural references may acquire affective significance through identity and shared meanings (Bassano et al., 2019; Herff et al., 2021). Personal memories and imagined experiences may similarly connect the song with emotionally meaningful aspects of the listener’s life (Juslin et al., 2014; Eskine et al., 2020). Thus:

H2a. Place imagery is positively associated with emotional experience.

H2b. Local cultural meaning is positively associated with emotional experience.

H2c. Associative experience is positively associated with emotional experience.

Research on mediated information and destination evaluation provides grounds for examining relational and behavioral associations (Jalilvand and Samiei, 2012; Guo and Pesonen, 2022). Favorable judgments may support willingness to visit and provide reasons to recommend or share destination-related content. Evaluations carrying personal significance may also relate to attachment. Accordingly:

H3a. Cognitive evaluation is positively associated with place attachment.

H3b. Cognitive evaluation is positively associated with visit intention.

H3c. Cognitive evaluation is positively associated with sharing intention.

Emotional involvement may connect the listening experience with psychological closeness to the represented place, consistent with research on tourism emotions and attachment (Prayag et al., 2017; Ramkissoon, 2023). Although musical responses vary across listeners and contexts (Vuoskoski and Eerola, 2011), emotional engagement may accompany place-related bonds, including those grounded in prior experience. Therefore:

H4. Emotional experience is positively associated with place attachment.

Place attachment gives a destination personal significance and may support willingness to seek or renew contact through travel (Prayag et al., 2017; Ramkissoon, 2023). Emotional involvement may also relate to visit intention through destination-related emotional salience and to sharing intention through personally meaningful expression. These proposed associations concern emotional involvement, acknowledging variation in the feelings elicited during listening (Vuoskoski and Eerola, 2011). Accordingly:

H5. Place attachment is positively associated with visit intention.

H6a. Emotional experience is positively associated with visit intention.

H6b. Emotional experience is positively associated with sharing intention.

Together, these arguments suggest that cognitive evaluation and emotional experience may relate to visit intention indirectly through place attachment. Favorable judgments and emotional involvement may each accompany personal bonds with the destination, which are associated with willingness to visit. Thus:

H7a. Cognitive evaluation has a positive indirect association with visit intention through place attachment.

H7b. Emotional experience has a positive indirect association with visit intention through place attachment.

Beyond these hypothesized associations, corresponding cognitive and affective pathways may differ in strength. As the theoretical arguments do not establish a consistent direction for these differences, asymmetry is examined through two exploratory research questions:

RQ1. For each symbolic cue—place imagery, local cultural meaning, and associative experience—does its association with cognitive evaluation differ in strength from its association with emotional experience?

RQ2. For each outcome—place attachment, visit intention, and sharing intention—do its associations with cognitive evaluation and emotional experience differ in strength?

Figure 1 presents the hypothesized relationships. The exploratory questions are assessed through direct comparisons of corresponding path coefficients.

**Figure 1:**
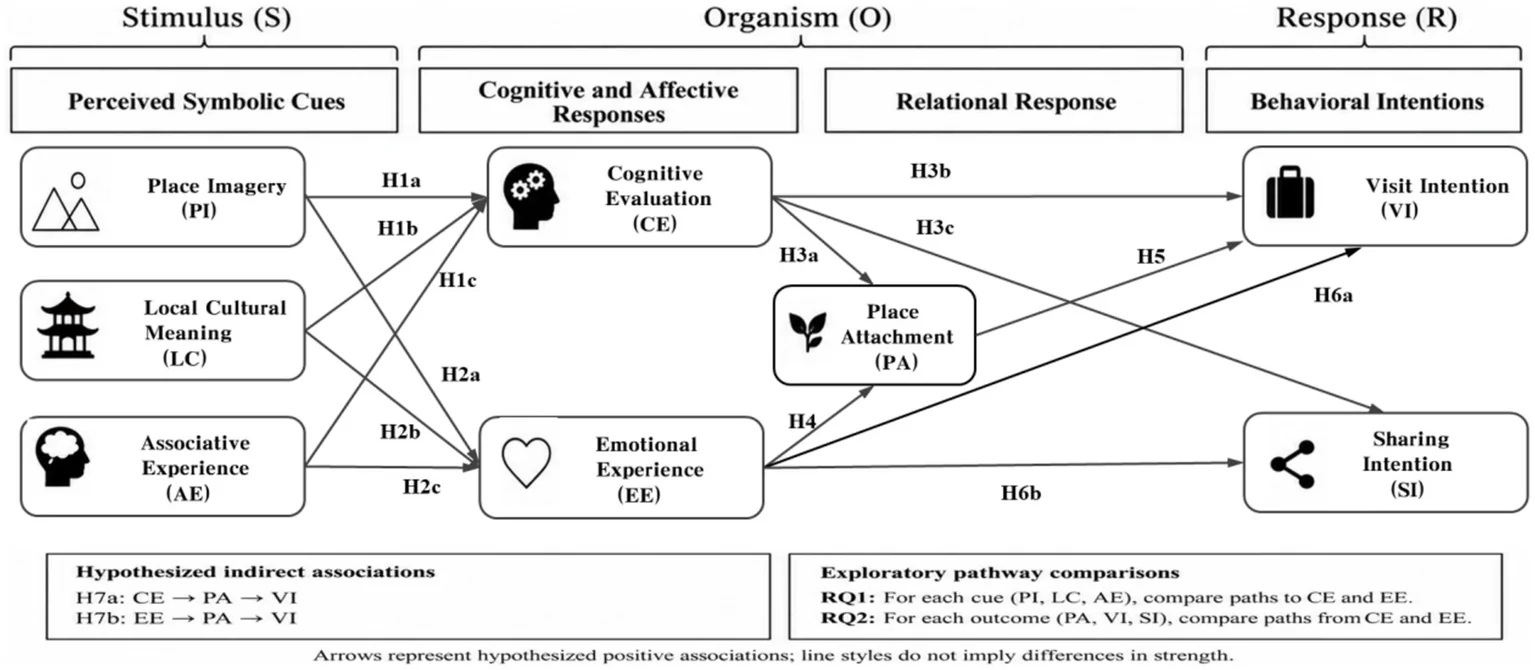
Cognitive–affective pathways under symbolic exposure. Arrows indicate positive hypotheses without implying relative strength. H7a–H7b concern mediation; RQ1–RQ2 compare pathway strengths.

## 3 Methodology

### 3.1 Data source and text preprocessing

This study used a sequential mixed-methods design combining user-generated content (UGC) analysis with survey-based structural modeling. Comments were collected from NetEase Cloud Music between January 2024 and August 2025. Fifteen destination-related songs were selected based on geographic diversity, cultural representativeness, and comment volume (Table 1). The initial corpus contained 60,601 comments, totaling approximately 1.85 million Chinese characters. Comments were obtained from publicly accessible pages without login restrictions; no personally identifiable information was retained in the analytical dataset.

**Table 1:** Characteristics and contextual relevance of the selected destination songs.

Song title	Release year	Destination	Artist	Genre/musical style	Emotional tone	Destination-related content
Mohe Ballroom	2020	Mohe	Liu Shuang	Folk-pop	Nostalgic and melancholic	Place-based storytelling associated with Mohe.
Lanzhou Lanzhou	2011	Lanzhou	Low Wormwood	Folk rock	Reflective and wistful	Local everyday life and cultural associations.
Beijing Beijing	2007	Beijing	Wang Feng	Rock ballad	Reflective and yearning	Urban experience and meanings associated with Beijing.
South of Colorful Clouds	2005	Yunnan	Xu Qianya	Pop with regional folk influences	Uplifting and yearning	Regional landscapes and cultural imagery.
Pearl of the Orient	1991	Hong Kong	Lo Ta-you	Pop ballad	Affectionate and solemn	Symbolic representations of Hong Kong and its urban identity.
Xiangjiang River	2021	Hunan	Lay Zhang feat. Kung Fu-Pen	Hip-hop/Changsha-dialect rap	Excitement and hometown pride	Changsha-dialect rap expressing local identity and hometown pride.
Changsha	2017	Changsha	C-BLOCK	Hip-hop/regional rap	Spirited and playful	Local urban life and place-specific references.
Guangdong Love Story	2017	Guangdong	Guangdong Yushen	Cantopop ballad	Melancholic and nostalgic	Personal relationships and everyday experiences situated in Guangdong.
Chengdu	2016	Chengdu	Zhao Lei	Folk-pop	Tender and wistful	Named streets and everyday urban settings.
Magical Jiuzhaigou	1999	Jiuzhaigou	Rongzhong Erjia	Pop with Tibetan musical influences	Reverent and uplifting	Scenic imagery associated with Jiuzhaigou.
Shanghai Bund	1980	Shanghai	Frances Yip	Cantopop	Dramatic and impassioned	Representations of Shanghai associated with a television narrative.
Crescent Bay	2007	Dunhuang	F.I.R.	Pop with Chinese musical influences	Romantic and yearning	Desert landscapes and cultural imagery associated with Dunhuang.
Return to Lhasa	2007	Lhasa	Zheng Jun	Rock with Tibetan musical influences	Exuberant and yearning	Place references and cultural imagery associated with Lhasa.
Summer in Xiamen	2011	Xiamen	LifeJourney Band	Indie pop	Lighthearted and relaxed	Coastal scenery and local urban settings.
West Lake	2008	Hangzhou	Miserable Faith	Folk rock	Contemplative and wistful	Scenic and cultural imagery associated with West Lake.

English titles are provided for identification. Genre and emotional tone are descriptive characterizations, not measures of listeners’ responses.

Preprocessing used Python-based tools, including Jieba and SnowNLP, to remove duplicate, excessively short, and irrelevant comments, followed by segmentation, stop-word and symbol removal, synonym standardization, and place-name normalization (Guo et al., 2017; Maier et al., 2018). The resulting corpus comprised 57,228 comments for LDA, whose themes informed survey construct contextualization.

### 3.2 Exploratory text analysis

Before topic modeling, exploratory diagnostics were conducted to characterize the corpus’s semantic structure. Keyword frequencies were computed using Python, with representative high-frequency terms reported in Table 2. Frequent words were dominated by destination-related expressions (e.g., Yunnan, Beijing, Chengdu, West Lake, Lhasa), affective vocabulary (e.g., like, hope, remembering), and identity-related references (e.g., motherland, friends, university), indicating that destination-song discourse centers on destination imagery, emotion, and shared symbolic meanings. The word cloud (Figure 2) further shows clustering around spatial imagery, emotional expression, and autobiographical association, suggesting that destination songs function as symbolic media through which listeners construct place meanings under mediated exposure (Guo et al., 2017; Maier et al., 2018). Overall, the exploratory analysis identifies recurring semantic domains associated with cognition, affect, and behavioral orientation, providing an empirical basis for subsequent LDA modeling and SEM construct development.

**Table 2:** High-frequency words extracted from destination-song reviews.

Keyword	Freq.	Keyword	Freq.	Keyword	Freq.
This song	5,225	Hope	1,058	Anniversary	655
Yunnan	4,400	Place	1,049	Friends	630
Like	3,385	Xiamen	949	Lijiang	602
Beijing	2,285	Return	919	Music	599
Chengdu	1,559	First time	807	Return to	584
West Lake	1,533	Thinking of	687	Remember	576
Lhasa	1,488	City	681	Snow Mountain	566
Pleasant to listen to	1,303	Hong Kong	676	Childhood	565
Changsha	1,223	Shanghai	667	Every time	560
Lanzhou	1,203	Crying hard	667	Shanghai Beach	554

Frequencies are based on 57,228 UGC comments retrieved from NetEase Cloud Music.

**Figure 2:**
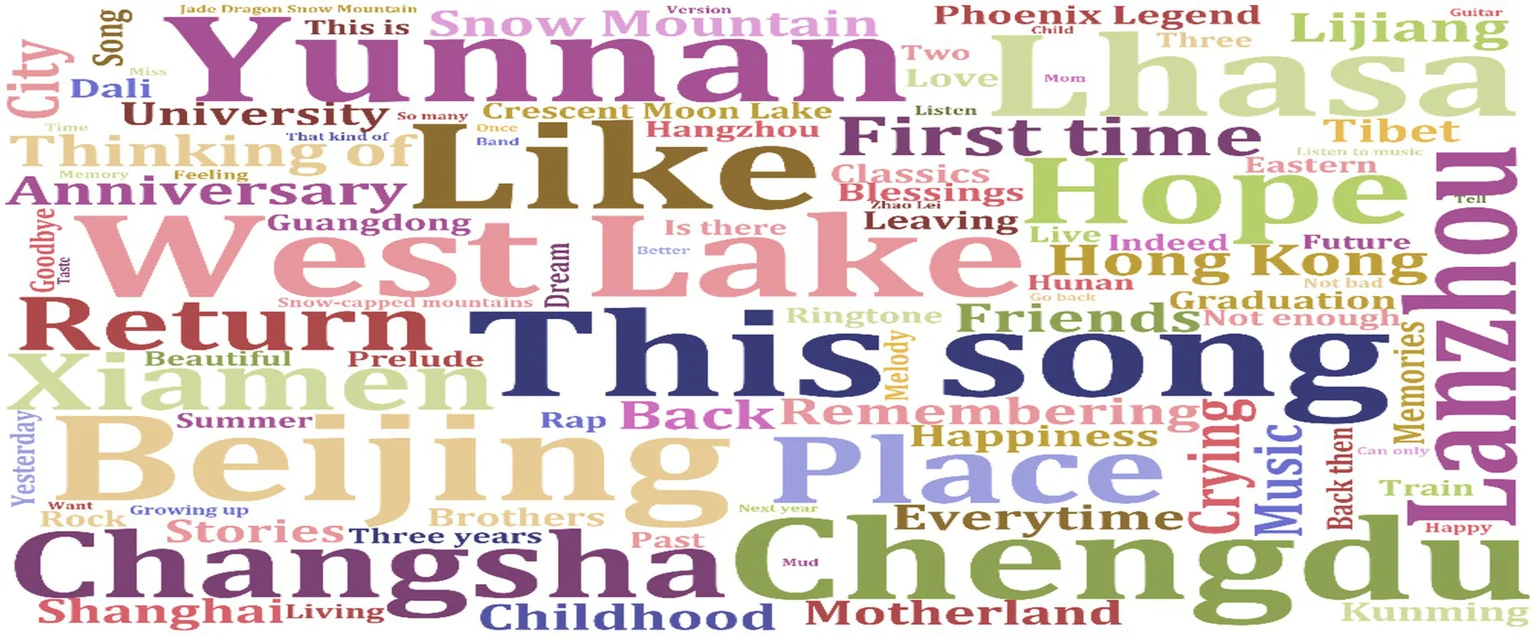
Word cloud of high-frequency terms in destination-song reviews. Larger words represent higher frequencies, emphasizing the dominance of spatial imagery, emotional expressions, and cultural symbols in listeners’ natural language responses.

### 3.3 Topic number selection and model estimation

LDA models with K ranging from 2 to 10 were compared using coherence and log-perplexity (Blei et al., 2003; Guo et al., 2017; Maier et al., 2018). Coherence was highest at K = 8 and remained close to this level at K = 9 (Figure 3). The nine-topic solution was retained for its semantic differentiation and interpretability. Topic prevalence, intertopic distances, and salient terms were visualized using pyLDAvis (Sievert and Shirley, 2014; Figure 4). The topic keywords subsequently informed theory-guided construct contextualization (Section 3.4).

**Figure 3:**
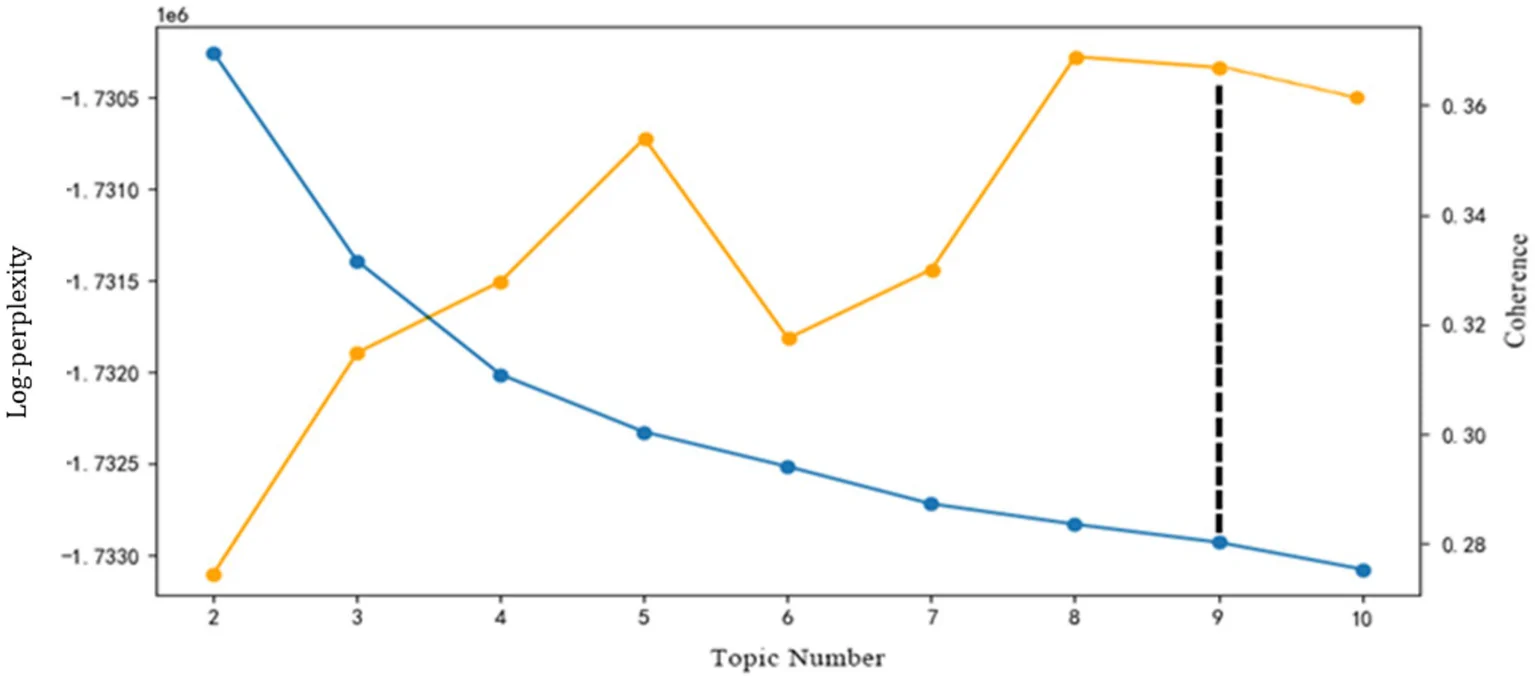
Log-perplexity and coherence across topic numbers.

**Figure 4:**
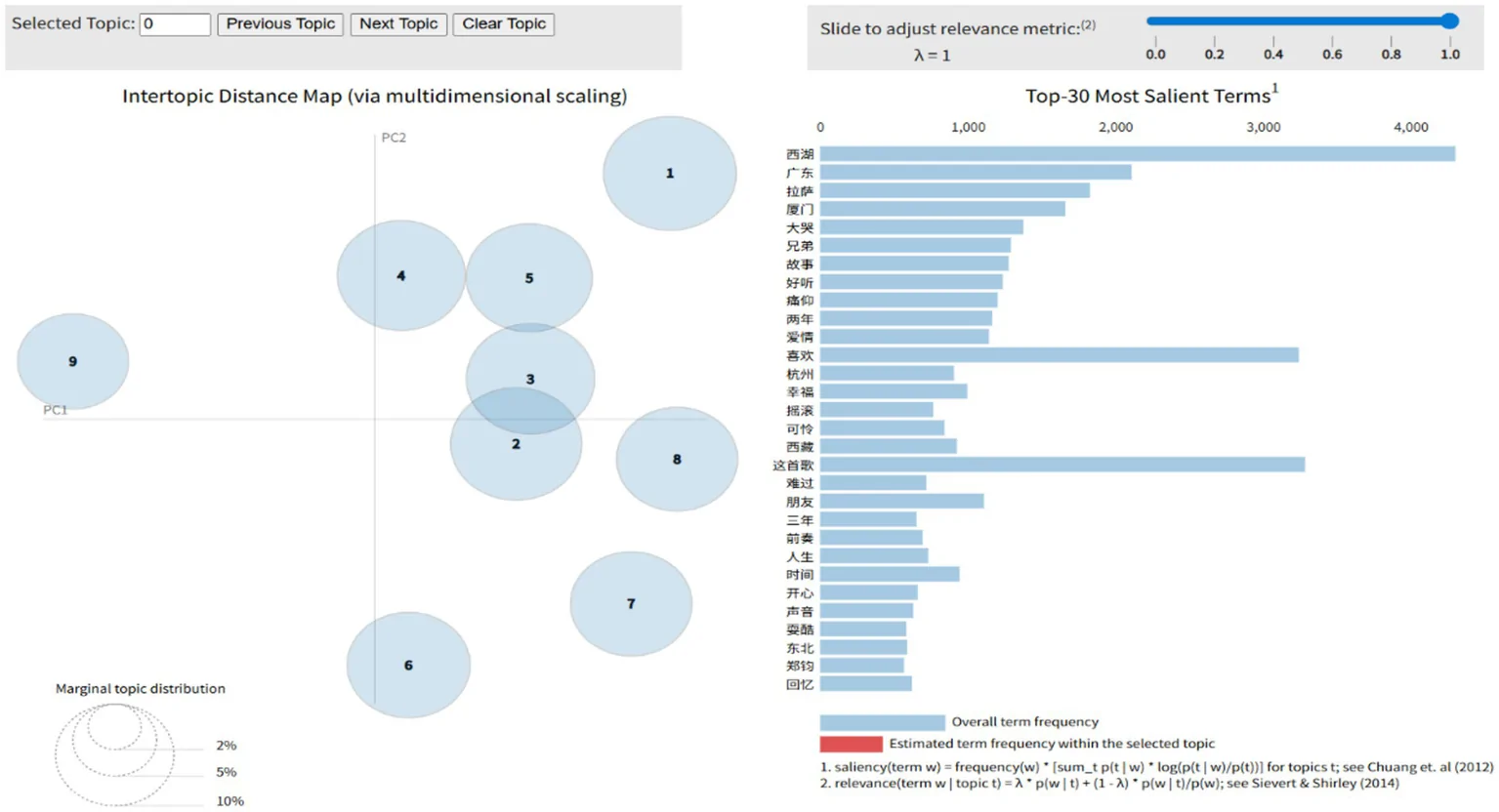
Intertopic distance map and salient term distribution of the final LDA model. Bubble size represents topic prevalence; distance indicates semantic distinctiveness.

### 3.4 Construct development and measurement design

LDA themes contextualized the survey constructs, while psychological theory guided their definitions and measurement (Guo and Pesonen, 2022; Pop et al., 2022). Three tourism experts established nine topic labels and their interpretive links to the constructs through three rounds of discussion. Two additional tourism experts independently assigned each topic to its most relevant construct using construct definitions, topic keywords, and linked comments, without seeing the proposed mappings or each other’s judgments. Their initial classifications agreed on all nine topics (100% agreement; Cohen’s κ = 1.00). Table 3 presents the interpretive links and illustrative comments. The original LDA topics remained unchanged, and these links were not treated as measurement equivalence.

**Table 3:** Theory-guided topic–construct links and illustrative comments.

Panel A. Topic–construct links			
LDA topic	Selected topic keywords	SEM construct	Interpretive link and theoretical basis
T5	West Lake; snow mountains; intro; guitar; soul	Place Imagery (PI)	Place and landscape references contextualize destination imagery; musical terms describe the listening context (Yin et al., 2020).
T4	rock; band; melody; Shanghai; Shenzhen; working	Local Cultural Meaning (LC)	Musical and urban references provide contextual cues for interpreting place-related cultural meanings (Bassano et al., 2019).
T1	Xiamen; college entrance examination; future; longing; postgraduate entrance examination	Associative Experience (AE)	Place references and life aspirations suggest personal associations relevant to musical imagination and autobiographical interpretation (Eskine et al., 2020; Margulis and Jakubowski, 2024).
T8	recommendation; friends; city; love; youth	Cognitive Evaluation (CE)	Recommendation-related keywords and a topic-linked comment expressing destination liking provide context for cognitive evaluation, which remains theoretically defined (Jalilvand and Samiei, 2012; Abubakar et al., 2017).
T2, T3, T9	T2: missing; hope; dislike. T3: recalling; high school; school. T9: sadness; memories; reminiscing.	Emotional Experience (EE)	Emotional expressions and autobiographical references jointly contextualize listening-related feelings; measurement addresses intensity and involvement (Juslin et al., 2014; Koelsch, 2014; López et al., 2024).
—	—	Place Attachment (PA)	Introduced from theory to represent psychological bonds with the destination, rather than derived from a distinct LDA topic (Chen et al., 2019; Ramkissoon, 2023).
T6	travel; returning home; companionship; dreams; yearning	Visit Intention (VI)	Travel and aspiration-related terms contextualize possible visitation interests; intention is operationalized through theory-guided measurement (Yin et al., 2020).
T7	music festival; concert; stranger; good night; graduation	Sharing Intention (SI)	Social references provide indirect context for interpersonal engagement; sharing intention is specified through communication research (Zhou et al., 2020).

Panel B. Illustrative topic-linked comments			
LDA topic	Comment excerpt (Chinese)	English translation	Interpretive relevance
T8	因为一首歌而喜欢上一座城市有生之年一定要来成都看一看	“A song made me like a city. I must visit Chengdu someday.”	Connects favorable destination evaluation with visit intention; illustrates related responses without establishing their equivalence.
T2	读书的时候听现在要当妈妈了这周就要和宝贝见面了希望一切顺利	“I listened to this while studying; now I am about to become a mother. I will meet my baby this week and hope everything goes smoothly.”	Connects listening with personal transitions and hope, providing autobiographical and emotional context.
T3	高中时第一次听到这首歌从此种下一颗种子现在坐在电子科大再听这首歌感慨万千	“I first heard this song in high school, and it planted a seed. Now, listening again at the University of Electronic Science and Technology of China, I feel deeply moved.”	Links school-related memories with present emotional involvement.
T9	每听一首当年喜欢的歌都会有一些回忆听这首回忆是年年末的内时候刚认识	“Every song I liked back then brings back memories. This one reminds me of the end of that year, when we had just met.”	Illustrates autobiographical recollection associated with listening; recollection alone does not establish emotional intensity or place attachment.

Excerpts are drawn from preprocessed comments and translated into English. Comments illustrate contextual meanings rather than establish topic–construct equivalence.

Keywords are selected translations from the LDA output. Topic–construct links reflect theory-guided interpretation, not measurement equivalence. PA was introduced from theory.

T5, T4, and T1 contextualized place imagery, local cultural meaning, and associative experience through place references, musical and urban narratives, and personal associations, respectively (Yin et al., 2020; Bassano et al., 2019; Eskine et al., 2020; Margulis and Jakubowski, 2024). T2, T3, and T9 remained separate topics but jointly informed the contextualization of emotional experience: their keywords concerned longing and hope, school-related recollections, and sadness and memories, respectively. These themes provided emotional and autobiographical context, while theory defined emotional experience as listening-related intensity and involvement (Juslin et al., 2014; Koelsch, 2014; López et al., 2024).

T8 provided indirect recommendation-related context for cognitive evaluation, which was theoretically defined as destination-focused judgment (Jalilvand and Samiei, 2012; Abubakar et al., 2017). Place attachment was introduced from theory to capture psychological bonds with the destination, including existing bonds from prior visits (Chen et al., 2019; Ramkissoon, 2023). T6 and T7 contextualized travel aspirations and social engagement, while theory guided the measurement of visit and sharing intentions (Zhou et al., 2020; Yin et al., 2020). Table 3 summarizes these interpretive links (Ali et al., 2022).

### 3.5 Measurement design

Measurement items were developed from theoretical definitions and contextualized using LDA themes. Place imagery, local cultural meaning, and associative experience drew on destination image, cultural meaning, and music-related association research (Yin et al., 2020; Bassano et al., 2019; Eskine et al., 2020). Cognitive evaluation and emotional experience addressed destination judgments and listening-related emotional involvement (Jalilvand and Samiei, 2012; Abubakar et al., 2017; Juslin et al., 2014; Koelsch, 2014). Place attachment, visit intention, and sharing intention addressed place-related bonds and behavioral intentions (Chen et al., 2019; Ramkissoon, 2023; Zhou et al., 2020; Yin et al., 2020). Table 4 reports the administered items and their theoretical foundations.

**Table 4:** Measurement constructs, items, and theoretical sources.

Construct	Code	Measurement item	Theoretical basis
Place Imagery (PI)	PI1	This song allows me to imagine the destination’s appearance even without visiting it.	Yin et al. (2020); Wei et al. (2023)
	PI2	This song helps me form a clear and vivid mental picture of the destination.	Yin et al. (2020)
	PI3	The scenes described in this song stimulate concrete imagination about the destination.	Wei et al. (2023)
	PI4	This song enhances my psychological imagination of the destination environment and atmosphere.	Herff et al. (2021)
Local Cultural Meaning (LC)	LC1	This song conveys the destination’s unique cultural atmosphere.	Bassano et al. (2019); Lee and Bai (2016)
	LC2	This song allows me to perceive the destination’s local identity and cultural characteristics.	Lee and Bai (2016)
	LC3	The cultural elements in this song enhance my understanding of the destination culture.	Bassano et al. (2019)
	LC4	This song strengthens my perception of the destination’s cultural uniqueness.	Moreno-Lobato et al. (2023)
Associative Experience (AE)	AE1	This song reminds me of my own life experiences or personal stories.	Eskine et al. (2020); Margulis and Jakubowski (2024)
	AE2	This song reminds me of certain people or past experiences.	Eskine et al. (2020)
	AE3	This song connects the destination with my own emotions.	Margulis and Jakubowski (2024)
	AE4	This song creates a feeling of imagining myself being in the destination.	McAuley et al. (2021)
Cognitive Evaluation (CE)	CE1	Based on this song, I consider the destination attractive.	Jalilvand and Samiei (2012); Abubakar et al. (2017)
	CE2	This song improves my overall evaluation of the destination.	Jalilvand and Samiei (2012)
	CE3	This song makes the destination worth considering for a future trip.	Abubakar et al. (2017)
	CE4	The symbolic meanings in this song increase my positive impression of the destination.	Guo and Pesonen (2022)
Emotional Experience (EE)	EE1	I experience clear emotional reactions while listening to this song.	Juslin et al. (2014); Koelsch (2014)
	EE2	This song creates emotional involvement for me.	Koelsch (2014)
	EE3	This song immerses me in its emotional atmosphere.	Juslin et al. (2014)
	EE4	This song evokes short-term but intense emotions.	Juslin et al. (2014)
Place Attachment (PA)	PA1	I feel emotionally connected to the destination described in this song.	Chen et al. (2019); Ramkissoon (2023)
	PA2	This destination holds personal meaning for me.	Ramkissoon (2023)
	PA3	I feel psychologically close to this destination.	Chen et al. (2019)
	PA4	I can imagine forming an emotional relationship with this destination.	Lu et al. (2022)
Visit Intention (VI)	VI1	After listening to this song, I would like to visit the destination in the future.	Yin et al. (2020); Zhou et al. (2020)
	VI2	This song increases my intention to visit the destination.	Yin et al. (2020)
	VI3	Compared with other destinations, I would be more willing to consider visiting this place.	Zhou et al. (2020)
	VI4	I would include this destination in my future travel plans.	Yin et al. (2020)
Sharing Intention (SI)	SI1	I would share this song or destination-related content on social media.	Zhou et al. (2020); Abubakar et al. (2017)
	SI2	I would recommend this song or destination to friends or family.	Abubakar et al. (2017)
	SI3	I would discuss topics related to this destination online.	Zhou et al. (2020)
	SI4	I would repost, comment on, or interact with destination-related content associated with this song.	Dogaru et al. (2024)

Items were developed with reference to the cited literature. English wording translates the administered Chinese items.

All items used five-point Likert scales (1 = strongly disagree; 5 = strongly agree). Expert review provided qualitative support for content validity, followed by a pilot survey with 150 participants. Item analysis and participant feedback guided revisions before the main survey; pilot responses were excluded from the main analysis. The questionnaire was developed in Chinese, and its English translation was checked through back-translation by two bilingual researchers.

### 3.6 Survey procedure and analytical strategy

Data were collected through Wenjuanxing from late October to December 2025. Invited members participated voluntarily and were randomly assigned one of 15 destination-related songs via a link. They were instructed to listen in full and immediately complete the questionnaire; repeat listening was permitted, but listening environments were neither controlled nor recorded. Eligibility required prior exposure to the assigned song, without a minimum familiarity level. Responses with missing values, incomplete listening, questionnaire completion times below 60 s, straight-line responses, or inconsistent screening answers were excluded. Of 575 questionnaires, 472 were retained (82.09%); 87.3% of respondents were moderately or very familiar with the song, and 91.7% had previously visited the destination (Table 5).

**Table 5:** Respondent characteristics and prior exposure (N = 472).

Characteristic and category	*n*	%
Gender		
Male	244	51.7
Female	228	48.3
Age		
18–25 years	79	16.7
26–35 years	169	35.8
36–45 years	119	25.2
46–55 years	59	12.5
≥56 years	46	9.7
Education		
High school or below	108	22.9
Junior college	107	22.7
Bachelor’s degree	173	36.7
Master’s degree	58	12.3
Doctoral degree or above	26	5.5
Occupation		
Student	79	16.7
Corporate employee	195	41.3
Public-sector employee^b^	115	24.4
Freelancer	37	7.8
Retired	46	9.7
Annual travel frequency		
0–1 trips	123	26.1
2–3 trips	213	45.1
4–5 trips	83	17.6
≥6 trips	53	11.2
Destination media exposure^a^		
Never	33	7.0
Occasionally	37	7.8
Sometimes	135	28.6
Often	166	35.2
Very often	101	21.4
Music-listening frequency		
Rarely	42	8.9
Occasionally	46	9.7
Sometimes	126	26.7
Often	169	35.8
Daily	89	18.9
Main music platform		
NetEase Cloud Music	145	30.7
QQ Music	162	34.3
Spotify	65	13.8
Apple Music	100	21.2
Song familiarity		
Heard but not familiar	60	12.7
Moderately familiar	240	50.8
Very familiar	172	36.4
Prior destination visits		
Never	39	8.3
Once	314	66.5
Twice or more	119	25.2

Percentages may not sum to 100% because of rounding.

a Frequency of exposure to destination information through music, short videos, or social media.

b Civil servants and public institution employees.

Anonymity and separate construct sections were used to reduce common method bias. Harman’s first factor explained 32.4% of the variance, although this does not rule out such bias. Structural paths and indirect effects were assessed in SmartPLS 4 using 5,000 bootstrap resamples. Exploratory pathway comparisons used 5,000 paired bootstrap resamples in Python (seed = 20,260,913), with full model re-estimation and two-sided 95% percentile confidence intervals for coefficient differences.

## 4 Results

### 4.1 Measurement model assessment

The measurement model showed satisfactory reliability and convergent validity (Hair et al., 2022). Outer loadings ranged from 0.764 to 0.867, Cronbach’s α from 0.818 to 0.872, composite reliability from 0.880 to 0.912, and AVE from 0.648 to 0.722 (Table 6). HTMT values ranged from 0.359 to 0.558, and the square roots of AVE exceeded the corresponding inter-construct correlations, supporting discriminant validity (Tables 7, 8; Henseler et al., 2015). Indicator-level VIF values ranged from 1.569 to 2.257.

**Table 6:** Reliability and convergent validity.

Construct	Item	Loading	Cronbach’s α	CR	AVE	VIF
PI	PI1	0.833	0.818	0.880	0.648	1.834
	PI2	0.794				1.624
	PI3	0.764				1.569
	PI4	0.826				1.868
LC	LC1	0.851	0.857	0.903	0.700	2.156
	LC2	0.855				2.148
	LC3	0.836				1.941
	LC4	0.804				1.771
AE	AE1	0.850	0.847	0.897	0.686	2.091
	AE2	0.801				1.740
	AE3	0.848				2.014
	AE4	0.814				1.793
CE	CE1	0.840	0.852	0.900	0.694	1.996
	CE2	0.783				1.696
	CE3	0.851				2.150
	CE4	0.855				2.052
EE	EE1	0.807	0.834	0.889	0.666	1.683
	EE2	0.814				1.749
	EE3	0.806				1.848
	EE4	0.838				1.941
PA	PA1	0.828	0.872	0.912	0.722	1.965
	PA2	0.867				2.257
	PA3	0.846				2.126
	PA4	0.858				2.160
VI	VI1	0.838	0.846	0.897	0.684	1.976
	VI2	0.812				1.858
	VI3	0.829				1.881
	VI4	0.831				1.848
SI	SI1	0.772	0.825	0.884	0.657	1.582
	SI2	0.827				1.843
	SI3	0.801				1.702
	SI4	0.840				1.981

Loadings are standardized outer loadings. CR = composite reliability (ρ_c); AVE, average variance extracted; VIF, indicator-level variance inflation factor. PI, place imagery; LC, local cultural meaning; AE, associative experience; CE, cognitive evaluation; EE, emotional experience; PA, place attachment; VI, visit intention; SI, sharing intention.

**Table 7:** Discriminant validity (HTMT).

Construct	PI	LC	AE	CE	EE	PA	VI	SI
PI	-							
LC	0.482	-						
AE	0.400	0.415	-					
CE	0.558	0.460	0.359	-				
EE	0.420	0.424	0.479	0.426	-			
PA	0.459	0.383	0.388	0.401	0.449	-		
VI	0.485	0.519	0.423	0.453	0.485	0.406	-	
SI	0.510	0.501	0.428	0.434	0.555	0.463	0.485	

All HTMT values are below the recommended threshold of 0.85. Construct abbreviations are defined in Table 6.

**Table 8:** Discriminant validity (Fornell–Larcker criterion).

Construct	PI	LC	AE	CE	EE	PA	VI	SI
PI	0.805							
LC	0.404	0.837						
AE	0.334	0.354	0.828					
CE	0.468	0.393	0.305	0.833				
EE	0.352	0.361	0.405	0.363	0.816			
PA	0.387	0.333	0.333	0.348	0.386	0.850		
VI	0.403	0.440	0.357	0.387	0.413	0.349	0.827	
SI	0.417	0.423	0.359	0.366	0.463	0.395	0.405	0.810

Diagonal values represent the square roots of AVE. Construct abbreviations are defined in Table 6.

### 4.2 Structural model and hypothesis testing

Inner VIF values ranged from 1.152 to 1.278, indicating no substantial collinearity among the predictors. The model yielded SRMR = 0.081 and NFI = 0.851. R^2^ values were 0.280 for cognitive evaluation, 0.245 for emotional experience, 0.199 for place attachment, 0.258 for visit intention, and 0.259 for sharing intention. All hypothesized direct paths were positive and significant (*p* < 0.001, except AE → CE: *p* = 0.012), with f^2^ values ranging from 0.015 to 0.170 (Figure 5; Table 9).

**Figure 5:**
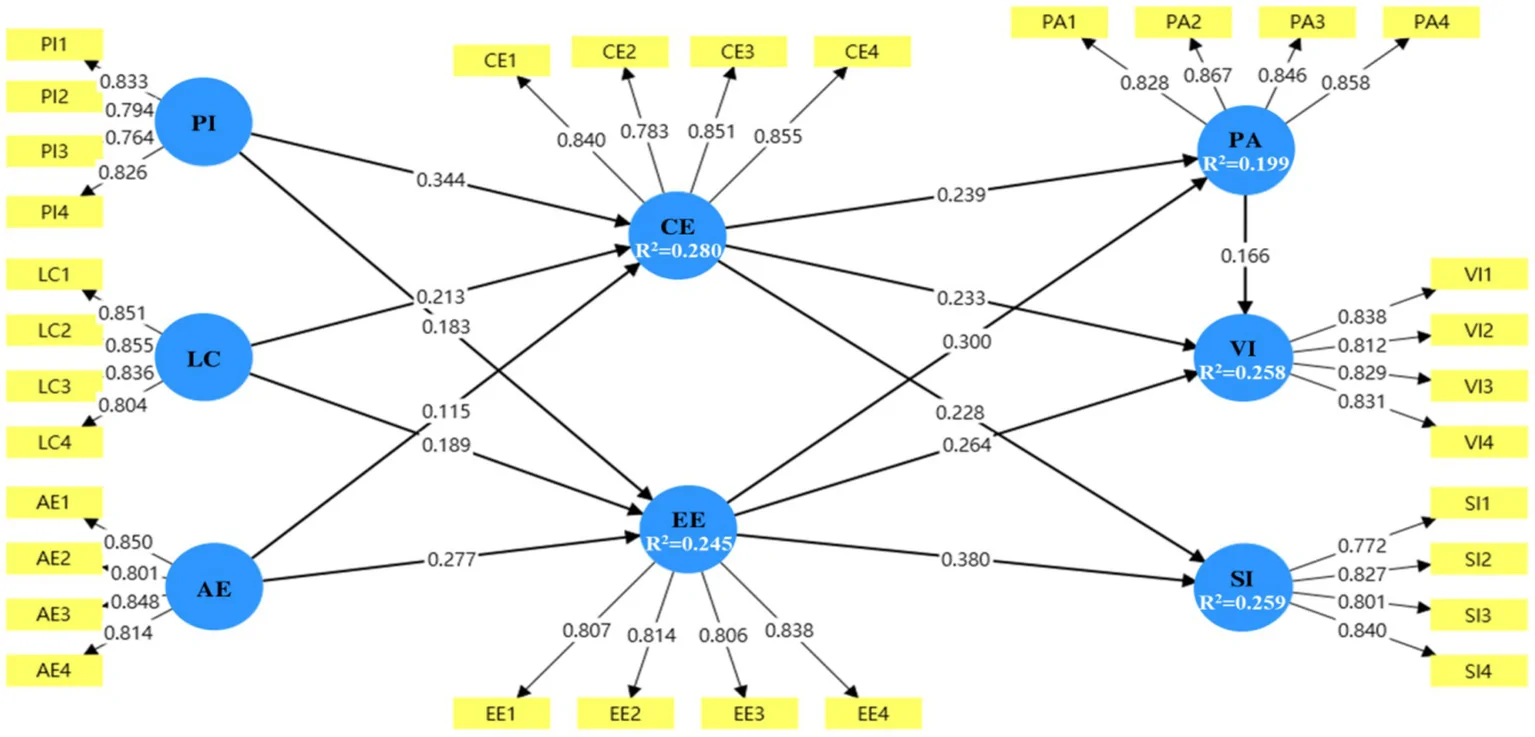
Structural model results with standardized path coefficients and explained variance (R^2^). Values on arrows are standardized path coefficients, and values within endogenous constructs are R2. All displayed paths are statistically significant (*p* < 0.05). PI, place imagery; LC, local cultural meaning; AE, associative experience; CE, cognitive evaluation; EE, emotional experience; PA, place attachment; VI, visit intention; SI, sharing intention.

**Table 9:** Structural model results and hypothesis testing.

Hypothesis	Structural path	*β*	*t*-value	*p*-value	f^2^	Result
H1a	PI → CE	0.344	7.469	<0.001	0.131	Supported
H1b	LC → CE	0.213	4.466	<0.001	0.049	Supported
H1c	AE → CE	0.115	2.526	0.012	0.015	Supported
H2a	PI → EE	0.183	3.841	<0.001	0.035	Supported
H2b	LC → EE	0.189	3.575	<0.001	0.037	Supported
H2c	AE → EE	0.277	5.952	<0.001	0.085	Supported
H3a	CE → PA	0.239	5.179	<0.001	0.062	Supported
H3b	CE → VI	0.233	4.993	<0.001	0.060	Supported
H3c	CE → SI	0.228	4.616	<0.001	0.061	Supported
H4	EE → PA	0.300	6.262	<0.001	0.097	Supported
H5	PA → VI	0.166	3.548	<0.001	0.030	Supported
H6a	EE → VI	0.264	5.554	<0.001	0.075	Supported
H6b	EE → SI	0.380	8.121	<0.001	0.170	Supported

β denotes the standardized path coefficient from the original sample; statistical significance was assessed using 5,000 bootstrap resamples. f^2^ denotes the effect size. Construct abbreviations are defined in Table 6.

Place imagery had the largest estimated association with cognitive evaluation (*β* = 0.344), whereas associative experience had the largest with emotional experience (*β* = 0.277). Cognitive evaluation and emotional experience were positively associated with place attachment (*β* = 0.239 and 0.300), visit intention (*β* = 0.233 and 0.264), and sharing intention (*β* = 0.228 and 0.380), respectively. Place attachment was also positively associated with visit intention (*β* = 0.166).

### 4.3 Exploratory tests of pathway asymmetry

Exploratory paired-bootstrap comparisons showed that place imagery was more strongly associated with cognitive evaluation than with emotional experience (Δ*β* = 0.161, 95% CI [0.050, 0.273]), whereas associative experience was more strongly associated with emotional experience than with cognitive evaluation (Δ*β* = 0.163, 95% CI [0.051, 0.273]). The corresponding difference for local cultural meaning was not significant. Differences between cognitive and emotional paths to place attachment, visit intention, and sharing intention were also not significant (Table 10). These results support asymmetry in selected antecedent pathways.

**Table 10:** Exploratory comparisons of cognitive and affective pathways.

Path comparison	Δβ	Bootstrap SE	95% percentile CI
PI → CE − PI → EE	0.161	0.057	[0.050, 0.273]
LC → CE − LC → EE	0.025	0.064	[−0.099, 0.150]
AE → EE − AE → CE	0.163	0.056	[0.051, 0.273]
EE → PA − CE → PA	0.061	0.082	[−0.098, 0.224]
EE → VI − CE → VI	0.031	0.077	[−0.118, 0.182]
EE → SI − CE → SI	0.153	0.085	[−0.014, 0.321]

Δβ is the first coefficient minus the second, using unrounded estimates. Two-sided 95% percentile CIs were obtained from 5,000 paired bootstrap resamples with full model re-estimation.

### 4.4 Indirect effects

Cognitive evaluation and emotional experience were indirectly associated with visit intention through place attachment (*β* = 0.040, 95% CI [0.016, 0.072]; *β* = 0.050, 95% CI [0.021, 0.086]), supporting H7a and H7b, respectively. Their direct associations remained significant, consistent with partial mediation. Exploratory analyses identified positive indirect associations for AE → EE → PA → VI (*β* = 0.014), AE → EE → SI (*β* = 0.106), and PI → CE → PA (*β* = 0.082), with all confidence intervals excluding zero (Table 11). These findings do not establish temporal or causal ordering.

**Table 11:** Hypothesized and selected exploratory indirect effects

Hypothesis	Indirect path	β	Bootstrap SE	*t*-value	*p*-value	95% bootstrap CI
H7a	CE → PA → VI	0.040	0.014	2.770	0.006	[0.016, 0.072]
H7b	EE → PA → VI	0.050	0.017	2.909	0.004	[0.021, 0.086]
—	AE → EE → PA → VI	0.014	0.006	2.452	0.014	[0.005, 0.027]
—	AE → EE → SI	0.106	0.023	4.546	<0.001	[0.066, 0.155]
—	PI → CE → PA	0.082	0.020	4.016	<0.001	[0.046, 0.126]

β = specific indirect effect; SE = bootstrap standard error; CI = confidence interval; — = exploratory analysis. Construct abbreviations are defined in Table 6.

### 4.5 Predictive relevance

PLSpredict yielded positive *Q*^2^_predict_ values for all constructs and indicators (Shmueli et al., 2019). PLS-SEM produced lower RMSE than the linear-model benchmark for all CE and EE indicators, two PA indicators, and three SI indicators, but none of the VI indicators. CVPAT showed lower prediction loss than the indicator-average benchmark across constructs and overall (all *p* < 0.001). Compared with the linear model, loss was significantly lower only for CE (*p* = 0.001) and EE (*p* = 0.036), with no significant overall difference (*p* = 0.465; Table 12).

**Table 12:** Predictive performance assessed using PLSpredict and CVPAT.

Construct	*Q*^2^_predict_ (LV)	*Q*^2^_predict_ (MV range)	Lower RMSE (n/4)	ΔLoss vs IA	*p*(IA)	ΔLoss vs LM	p(LM)
CE	0.265	0.145–0.206	4/4	−0.260	<0.001	−0.031	0.001
EE	0.232	0.098–0.177	4/4	−0.204	<0.001	−0.023	0.036
PA	0.175	0.117–0.134	2/4	−0.182	<0.001	0.005	0.816
VI	0.219	0.136–0.159	0/4	−0.217	<0.001	0.013	0.544
SI	0.222	0.126–0.158	3/4	−0.207	<0.001	0.002	0.920
Overall	—	—	—	−0.214	<0.001	−0.007	0.465

LV = construct level; MV = indicator level; LM = linear model; IA = indicator average. RMSE counts compare PLS-SEM with LM; negative ΔLoss favors PLS-SEM.

## 5 Discussion

### 5.1 Cognitive, affective, and relational pathways

The findings address how feelings during destination-song listening relate to evaluations of, bonds with, and intended responses toward the represented place. Pathway comparisons showed that place imagery was more strongly associated with cognitive evaluation, whereas associative experience was more strongly associated with emotional experience. However, cognitive–affective differences in the paths to attachment, visit intention, and sharing intention were not statistically supported. Asymmetry therefore concerns selected antecedent pathways. The PI → CE → PA indirect association further links place imagery with attachment through evaluation, consistent with accounts of narrative imagination and meaning construction in music (McAuley et al., 2021; Margulis and Jakubowski, 2024; Hashim et al., 2023).

Both cognitive evaluation and emotional experience were indirectly associated with visit intention through place attachment, identifying a shared relational pathway. The additional AE → EE → PA → VI association connects personal associations with this pathway, consistent with music psychology accounts of memory, imagery, and emotional engagement (Juslin et al., 2014; Koelsch, 2014; Warrenburg, 2020). Because 91.7% of respondents had previously visited the destination, this small indirect effect may reflect engagement with existing place-related experiences rather than newly formed attachment. Such an interpretation remains tentative and recognizes the contextual dependence of musical responses (Vuoskoski and Eerola, 2011).

The AE → EE → SI association also links personal associations with willingness to share songs or destination-related content through emotional experience (Zhou et al., 2020; Dogaru et al., 2024). This extends the analysis beyond visit intention to communicative engagement, without equating sharing with destination promotion or actual behavior. Together, the findings specify how listening-related emotion and destination evaluation connect with place attachment and distinct behavioral intentions.

### 5.2 Theoretical contributions and explanatory boundaries

This study elaborates the Stimulus–Organism–Response (SOR) framework by distinguishing the antecedents of cognitive and emotional responses from their relational connections with behavioral intentions. Building on tourism research on emotions, evaluations, attachment, and intentions (Prayag et al., 2017; Ramkissoon, 2023; Yeh et al., 2012), it shows that differentiated cognitive–affective associations can coexist with a shared attachment pathway. The contribution lies in locating where asymmetry occurs within the model, without assuming that either cognition or emotion consistently dominates destination-related responses.

The study also clarifies symbolic exposure as a mediated encounter that can draw on prior firsthand experience. Embodied tourism research emphasizes environmental interaction (Fan et al., 2023; Chan et al., 2025), while music research highlights imagination, memory, and narrative interpretation (McAuley et al., 2021; Margulis and Jakubowski, 2024; Herff et al., 2021). Together, these perspectives suggest that destination songs may engage meanings grounded in previous visits alongside imagined experiences, consistent with the personal and contextual nature of musical responses (Vuoskoski and Eerola, 2011; López et al., 2024; Klempe, 2017). The distinction between exposure mode and experiential history provides an explanatory boundary; whether prior experience moderates these pathways remains untested.

Methodologically, the study connects UGC analysis with survey measurement (Guo and Pesonen, 2022; Pop et al., 2022; Ali et al., 2022). Expert interpretation of LDA themes contextualizes theoretically defined constructs, while the survey assesses their measurement properties and associations. This approach links semantic exploration with psychological measurement without treating topic labels as validated constructs.

### 5.3 Managerial implications

The three indirect pathways suggest practical options for destination management organizations (DMOs). PI → CE → PA suggests pairing songs with identifiable landscapes, local stories, and brief explanations of cultural references to help listeners interpret the destination’s appeal (Jalilvand and Samiei, 2012; Yin et al., 2020; Abbasi et al., 2023). AE → EE → SI suggests inviting personal memories and stories as material for voluntary sharing (Dogaru et al., 2024; Zhou et al., 2020). AE → EE → PA → VI further suggests connecting these recollections with opportunities to revisit meaningful places, particularly for previous visitors (Chen et al., 2019; Ramkissoon, 2023; Chan et al., 2025).

For example, a DMO could pair a destination song with an image of the place and a brief explanation of a culturally specific lyric. A prompt such as “What memory does this song bring back?” could invite personal stories, while a linked page provides walking routes, transport information, and local events. This approach connects destination interpretation and emotional engagement with practical travel information. Because the observed associations do not establish that such campaigns strengthen attachment or increase visitation, these proposals require field testing. Comments and shares should be tracked separately from itinerary clicks and verified visits.

### 5.4 Limitations and future research

The cross-sectional, self-report design limits causal and temporal inference and remains susceptible to common method bias. Most respondents had previously visited the destination, so attachment may reflect existing bonds. Longitudinal and experimental studies could distinguish immediate listening responses from changes in attachment. Musical genre and emotional tone were described but not statistically controlled, and emotional experience captured intensity and involvement rather than valence. Future studies could control or manipulate these characteristics and compare listeners with different visitation histories. Comparisons across media formats, incorporating construal level and psychological distance, could clarify the boundaries of cognitive–affective associations. Research should also distinguish song sharing from destination-content sharing and assess observed behavior alongside intentions. The Chinese destination-song context, single-platform UGC source, and uneven predictive performance, particularly for visit intention, warrant validation across samples and settings.

## 6 Conclusion

This study identifies differentiated cognitive and affective associations in music-mediated destination perception. Place imagery was more strongly associated with cognitive evaluation, whereas associative experience was more strongly associated with emotional experience. Both responses were indirectly associated with visit intention through place attachment, but their corresponding paths to attachment and behavioral intentions did not differ significantly. These findings refine the SOR framework by locating asymmetry in selected antecedent pathways and identifying attachment as a shared relational link. Symbolic exposure may engage both imagined and previously experienced place meanings, without establishing causal or temporal ordering.

## Data Availability

The raw data supporting the conclusions of this article will be made available by the authors, without undue reservation.
